# Roasting temperature and fat type influence cholesterol oxidation products, fatty acid composition, physicochemical properties and sensory attributes of beef sausages

**DOI:** 10.1371/journal.pone.0322290

**Published:** 2025-04-25

**Authors:** Kazeem Dauda Adeyemi, Latifat Opeyemi Abdulkadir

**Affiliations:** Department of Animal Production, Faculty of Agriculture, University of Ilorin, PMB, Ilorin, Nigeria.; Prince of Songkla University, THAILAND

## Abstract

The impact of fat type (FT) and roasting temperature (RT) on oxysterols, physicochemical properties and sensory attributes of beef sausages were investigated. Beef sausages were formulated with either 20% Beef tallow (BT), Palm olein (PO) or Soybean oil (SO), and oven-cooked at either 180^o^C for 30 min or 240^o^C for 15 min. The BT, PO, and SO sausages had the highest (P<0.05) levels of saturated fatty acids (SFA), monounsaturated fatty acids (MUFA), and polyunsaturated fatty acids (PUFA), respectively. Roasting at 240°C increased PUFA, MUFA, and total cholesterol levels, and reduced SFA moisture, and fat levels (*P*<0.05). The FT × RT interaction was significant for oxysterols, instrumental color and cook loss. The SO-180 sausages had higher levels of 7-ketocholesterol, 5,6β-epoxy cholesterol, cholesta-3,5-dien-7-one, and total oxysterols, but these levels decreased significantly at 240°C. The BT sausages exhibited lower overall cholesterol oxidation, though 22R-hydroxycholesterol was elevated at 180°C, while the PO sausages showed intermediate oxysterol oxidation, with 7α-hydroxycholesterol increasing at 240°C (*P*<0.05). The SO sausages had higher TBARS compared to other sausages (*P*<0.05). The BT sausages had greater lightness and lower cook loss at 240°C, while redness increased in both BT and SO sausages at 240^o^C (*P*<0.05). The BT sausages had higher hardness and chewiness. The PO sausages had better taste scores than the BT sausages, with similar results to SO sausages, and both PO and SO sausages had higher appearance and overall acceptance scores than the BT sausages. Fat type and roasting temperature synergistically affect oxysterol levels and quality attributes of beef sausages, highlighting the importance of selecting suitable fats and roasting conditions to ensure safety, nutritional value, and sensory quality.

## 1. Introduction

Sausages are a popular meat product worldwide, valued for their convenience, flavor, and versatility [1, 2]. However, their nutritional composition, particularly the fat content, has raised concerns regarding their potential impact on human health [3, 4]. The type of fat used in sausage formulations plays a crucial role in determining the fatty acid (FA) profile, which in turn influences the nutritional quality, stability, and sensory attributes of the final product [5–8]. Different fats contain varying proportions of saturated (SFA), monounsaturated (MUFA), and polyunsaturated fatty acids (PUFA), each with distinct effects on human health and food stability [7–9]. While SFAs contribute to firmness and oxidative stability [10, 11], excessive intake is associated with cardiovascular risks [12, 13]. MUFAs are known to enhance juiciness and tenderness [10, 11] while offering potential cardiovascular benefits [14, 15]. On the other hand, PUFAs provide essential fatty acids and have been linked to various health benefits, including anti-inflammatory effects and improved lipid metabolism [16, 17]. However, PUFAs are more susceptible to oxidation, leading to the formation of hydroperoxides and aldehydes, which can compromise product quality and may pose health risks upon consumption [18–20]. Previous studies on meat products have shown that fat type influences oxidative stability, lipid degradation, and sensory perception [5–8], yet its impact in roasted beef sausages remains underexplored.

Thermal processing is often required to transform raw meat into consumable products [21, 22]. It ensures food safety by eliminating harmful pathogens and improves palatability by enhancing flavor, texture, and aroma [22–24]. In addition, it prolongs shelf life by reducing water content and inhibiting microbial growth [22,24,25]. Temperature is a crucial variable in thermal processing of meat [22–24]. The cooking temperature directly influences food safety, product quality, and shelf life [21,23]. Meat products are roasted or grilled at high temperatures to enhance flavor through the Maillard reaction, create a crispy exterior, and reduce cooking time [23,24,26]. However, high heat can lead to water loss, resulting in drier meat products, accelerate oxidation, and reduce nutritional quality [23,27]. In addition, it can cause the formation of harmful compounds like heterocyclic amines [22,28] and polycyclic aromatic hydrocarbons [29]. However, the relationships between fat type and roasting temperature on sausage quality remain less explored.

Animal-derived foods are rich in cholesterol [30], which undergoes oxidation during processing and storage when exposed to heat, light, oxygen, and catalysts, leading to the formation of cholesterol oxidation products (COPs) [31, 32]. COPs are considered more harmful to arterial cells than pure cholesterol and are directly associated with atherosclerosis and coronary heart diseases [31,33]. Furthermore, COPs inhibit cholesterol biosynthesis and reduce its dietary absorption [34]. As a result, COPs are recognized as cytotoxic, mutagenic, and carcinogenic [30–32]. Cooking methods [35–38] and the degree of fat saturation [39] can influence the type and quantity of COPs in meat products. However, the impact of cooking temperature, fat types, and their interaction on COPs has not been thoroughly investigated in roasted beef sausages. Understanding these effects on beef sausage quality is crucial for the meat processing industry, as it could affect both consumer health and product acceptability. This study aimed to evaluate the influence of fat type and roasting temperature on cholesterol oxidation products, fatty acids, textural profile, physicochemical properties, and sensory attributes of beef sausages.

## 2. Materials and methods

### 2.1. Raw materials

Six batches of beef Semitendinosus muscle (4.47 ± 0.43 kg per batch) and beef tallow were procured from a meat shop in Ilorin, Nigeria. Palm olein, soybean oil and other sausage ingredients were sourced from grocery stores in Ilorin, Nigeria.

### 2.2. Beef sausage preparation

Each batch of meat, which constituted a replicate, was processed into sausages separately. The beef was trimmed of external fat and connective tissue, diced into small pieces, and minced using a food processor (XM476831, Senbowe Electronics USA Inc, Arcadia, CA 91006, USA). The minced meat was then combined with all other ingredients, except for the fats. The mixture was divided into three portions, and each was mixed with either 20% soybean oil (SO), palm olein (PO), or beef tallow (BT), as detailed in Table 1. The sausage mixture was manually homogenized and rested for 4 h at 5±1°C. It was then portioned into 50 g samples, stuffed into a 20 mm diameter collagen casing (Haiaos Sausage Casing Co., Ltd, Zibo, China) using a plastic sausage stuffer, placed in silicon sausage molds, and roasted in an oven (WTC Binder FD53, Binder GmbH, Tuttlingen) at either 180°C for 30 min or 240°C for 15 min. Preliminary trials showed that sausages roasted at 180°C and 240°C reached a core temperature of 78±1°C within 30 and 15 min, respectively, explaining the selection of these roasting conditions. Six separate batches of beef sausages were prepared, with each batch considered a replicate. Each batch produced 144 sausages, with each weighing 50 g. Within a batch, each treatment combination had 24 sausages of 50 g each.

**Table 1:** Composition of beef sausage.

	Fat type		
Ingredient (%)	Beef tallow	Palm olein	Soybean oil
Beef	65.00	65.00	65.00
Soybean oil	0.00	0.00	20.00
Palm olein	0.00	20.00	0.00
Beef tallow	20.00	0.00	0.00
Corn starch	10.00	10.00	10.00
NaCl	1.00	1.00	1.00
Seasoning	1.00	1.00	1.00
*Capsicum chinense*	0.50	0.50	0.50
Ginger powder	0.50	0.50	0.50
Garlic powder	0.50	0.50	0.50
Onion powder	0.50	0.50	0.50
Ice	1.00	1.00	1.00

The treatment combinations are as follows: BF-180, Beef sausages containing 20% beef tallow and roasted at 180^o^C for 30 min; BF-240, Beef sausages containing 20% beef tallow and roasted at 240^o^C for 15 min; PO-180, Beef sausages containing 20% palm olein and roasted at 180^o^C for 30 min; PO-240, Beef sausages containing 20% palm olein and roasted at 240^o^C for 15 min; SO-180, Beef sausages containing 20% soybean oil and roasted at 180^o^C for 30 min; SO-240, Beef sausages containing 20% soybean oil and roasted at 240^o^C for 15 min.

### 2.3. Chemical composition

The proximate composition of beef sausages was determined using AOAC [40] methods within 20 min after roasting. Water content was measured by drying the samples at 105 °C until a constant weight was achieved. Crude protein was analyzed using the Kjeldahl method, with a nitrogen-to-crude protein conversion factor of 6.25. Crude fat was calculated based on weight loss after an 8-hour extraction with petroleum ether using a Soxhlet apparatus. Ash was determined by incinerating samples in a muffle furnace at 550 °C for 8 h.

### 2.4. Color, cook loss, and pH analyses

Color and pH assessments were carried on roasted beef sausages within 10 min after roasting. Instrumental color was measured using a pre-calibrated colorimeter (WR-10, Shenzhen, China; D65 illuminant, 10° observer, 8 mm aperture) to determine the CIE L*, a*, and b* values. Six readings were taken from the inner and outer points on each sample, and the average was recorded.

The pH was measured using a pH meter (MW102, Milwaukee® Instruments, Inc., Rocky Mount, NC, USA). Prior to measurement, the pH meter was calibrated according to the manufacturer’s instructions using standard buffer solutions with pH values of 4.01 and 7.01 at 25°C. The pH measurement was performed on a homogenate prepared by blending 2.5 g of the sample with 25 mL of distilled water. Cook loss was calculated based on the percentage difference in sample weight before and after roasting.

### 2.5. Texture analysis

Texture profile analysis of the roasted sausages was conducted at room temperature using a texture analyzer (TA-XT2i, Texture Technologies Corp., Scarsdale, NY), following the procedure outlined by Bourne [41]. A 25 kg load cell was used to calibrate the equipment, and the test involved two successive compression cycles with a 5-second interval between compressions. Samples, 2 inch thick and 1 inch in diameter, were compressed to 70% of their original height at a speed of 1 mm/s, using a P-35 probe (35 mm diameter, stainless steel). The parameters assessed were hardness, cohesiveness, gumminess, springiness, and chewiness. Gumminess was calculated by multiplying cohesiveness and hardness values. Chewiness was calculated by multiplying cohesiveness, springiness and hardness values.

### 2.6. Lipid oxidation and protein oxidation analyses

Lipid oxidation was assessed using the 2-thiobarbituric acid (TBA) method [42]. One gram of roasted beef sausage was mixed with 5 mL of a stock solution containing 0.03 M thiobarbituric acid (Sigma-Aldrich, St. Louis, MO, USA), 0.92 M trichloroacetic acid (Merck, Darmstadt, Germany), and 0.25 M HCl. After vortexing, the samples were heated in a boiling water bath for 10 min. Once cooled, the samples were centrifuged and filtered. The absorbance was then measured at 532 nm. TBARS values were calculated using the formular below:


$$TBARS\left(mgMDA/kgsample\right)=\frac{A\times V\times MV}{\varepsilon \times b\times W}$$

A= Absorbance of sample at 532 nm

V = Total volume of the reaction mixture (mL)

MW = Molecular weight of malondialdehyde (MDA) (72.06 g/mol)

ε = Molar extinction coefficient of MDA-TBA complex (156,000 M/cm)

b = Path length of the cuvette (1 cm)

W = Sample weight (g)

Protein oxidation was assessed by quantifying carbonyl groups through their reaction with DNPH (Sigma-Aldrich, St. Louis, MO, USA) to form hydrazones following the method of Levine et al. [43]. Briefly, 0.1 g of roasted beef sausage was incubated with 1.0 mL of 20 mM DNPH solution for 1 h. Proteins were precipitated by adding 20% (v/v) TCA (Merck, Darmstadt, Germany) and re-dissolved in the DNPH solution. The proteins were then precipitated again using 20% (v/v) TCA. The resulting protein pellet was washed three times with 125 μL of an ethanol/ethyl acetate (1:1) mixture and re-suspended in 125 μL of 6 M/L guanidine hydrochloride (Sigma-Aldrich, St. Louis, MO, USA). Absorbance was measured at 370 nm. The carbonyl content was calculated using the extinction coefficient for DNPH and expressed as mmol carbonyl/mg protein as follows:


$$Carbonylcontent=\frac{A\times DF\times V}{\varepsilon \times b\times W}$$

Where:

A = absorbance of the sample measured at 370 nm

DF = Dilution factor during sample preparation

V = Volume of sample after extraction,

*ε* = extinction coefficient for DNPH (22,000/M/cm)

b = The path length of cuvette (1 cm)

W = Weight of sample (g)

### 2.7. Fatty acid analysis

A Chloroform: methanol (2:1 v/v) mixture was used to extract the total lipids from fats and roasted sausage samples [44]. The extracted lipids were treated with 0.66 N KOH in methanol and 14% methanolic BF_3_ to obtain the fatty acid methyl esters (FAME) [45]. C21:0 (Sigma-Aldrich, Inc., St. Louis, Missouri, USA) was the internal standard. A gas chromatograph-mass spectrometer (GCMS-QP2010SE, Shimadzu, Japan) equipped with an SP®-2560 Capillary column (100 m × 0.25 mm × 0.20 μm, Supelco, Inc., Bellefonte, PA, USA) was used to accomplish the FAME separation. The column oven was set to operate at 100°C for 3 min, after which it was raised to 280°C at a rate of 5°C per min and maintained there for a further 20 min. The carrier gas was Helium, flowing at a rate of 1 mL/min (with a split ratio of 1:30) with a linear velocity of 44.3 cm/s and pressure of 11.604 psi. The detector’s temperature was kept at 280°C while the injector was set at 250°C. Based on the retention time and peak areas of established standards, sample peaks were identified and concentrations were calculated [46]. To calculate recoveries and correction factors for individual fatty acid tests, a reference FAME standard (Mix C4–24, 18919–1AMP; Supelco, Bellefonte, USA) was utilized.

### 2.8. Determination of total cholesterol

Total cholesterol in roasted beef sausages was determined using the spectrophotometric method described by Rudel and Morris [47]. A 1 g beef sausage was homogenized with 3 mL ethanol and 2 mL of 50% KOH. The homogenate was incubated in a water bath at 60°C for 15 min, then allowed to cool to room temperature (30 °C). A 5 mL hexane was added to the homogenate, followed by mixing for 30 s and 3 mL of deionized water was added, and the mixture was vortexed for 3 min and left to stand at 30 °C for 20 min to allow phase separation. A 2.5 mL of the upper hexane layer was carefully transferred to a clean glass tube, and the solvent was evaporated to dryness in a water bath at 50°C. The residue was reconstituted with 4 mL of o-phthalaldehyde reagent and left at 30 °C for 15 min. Subsequently, 2 mL of concentrated H_2_SO_4_ was slowly added, followed by thorough mixing and an additional 15 min incubation at 30 °C. Cholesterol standards (Sigma L-4646) were prepared following the method of Rudel and Morris [47] to obtain final concentrations of 0–100 μg cholesterol/mL. The absorbance of the samples and standards was measured at 550 nm using a spectrophotometer (Visible Spectrophotometer 721, Axiom Medical, U.K.). The total cholesterol content was determined from a standard curve using the formula:


$$Cholesterol\left(\frac{mg}{100g}\right)=\frac{\text{A sample}-\text{A blank}}{\text{A standard}-\text{A blank}}\times concentrationofstandard\times Dilutionfactor$$

Where:

A sample = Absorbance of the sample

A blank = Absorbance of the blank

A standard = Absorbance of the standard

Dilution factor accounts for sample preparation and extraction steps

### 2.9. Cholesterol oxides analysis

Cholesterol oxides were saponified following the direct sample saponification procedures described by Dionisi et al. [48] and extracted as described by Mariutti et al. [49]. Briefly, 2 g of roasted sausage was mixed with 6 mL of ethanol and 4 mL of 50% KOH, and the mixture was incubated in the dark at 27 °C for 22 h. After incubation, 5 mL of distilled water and 10 mL of hexane were added, and the mixture was vortexed. The hexane fraction was separated, and the extraction with 10 mL of hexane was repeated three times. The combined hexane extracts were subjected to solid-phase extraction (SPE) to isolate cholesterol oxides. The hexane extract was loaded onto a silica SPE cartridge (Sep-Pak, Waters, Milford, MA, USA) pre-conditioned with hexane. Cholesterol oxides were then eluted using hexane:2-propanol, 9:1 v/v. The purified eluate was collected and dried using a rotary evaporator. The residue was dissolved in 5 mL of hexane, transferred to a screw-top flask under a nitrogen atmosphere, diluted with 1 mL of mobile phase, and filtered through a 0.22 μm filter (Millipore, Maryland, MD, USA) prior to HPLC analysis. The chromatographic analysis of COPs was performed using a BIOBASE HPLC (EClassical 3200, Shandong, China) equipped with a D3210 UV–Vis detector. A 30 µL aliquot of the sample was injected, and separation was achieved under isocratic conditions on a 4.6 mm × 250 mm C18 reversed-phase column packed with 5 µm ultra-pure silica gel (Vertex). The flow rate of the mobile phase was 1 mL/min. Detection was carried out at 212 nm using UV–Vis spectroscopy. Data acquisition and processing were conducted using Biobase N2000 Chromatographic Data System software. COPs were identified by comparing the retention times and mass spectra of sample peaks with those of verified standards (Sigma-Aldrich, St. Louis, MO, USA). Quantification of oxysterols was performed using calibration curves generated from the respective standards [49].

### 2.10. Sensory analysis

The experimental procedure adhered to the guidelines approved (FERC/ANPR/2022/196) by the Ethical Review Committee, University of Ilorin, and informed written consent was obtained from all assessors. The sensory assessment was conducted on 25^th^ April 2023. Sensory evaluation was conducted by a consumer panel consisting of individuals familiar with sausage consumption, in accordance with the American Meat Science Association (AMSA) [50] guidelines. Sensory evaluation was conducted using a 9-point hedonic scale, where 9 = like extremely, 8 = like very much, 7 = like moderately, 6 = like slightly, 5 = neither like nor dislike, 4 = dislike slightly, 3 = dislike moderately, 2 = dislike very much, and 1 = dislike extremely. The sausages were served hot (55 °C). The sensory attributes assessed included taste, flavor, juiciness, appearance, tenderness, and overall acceptability. The panel consisted of 33 males and 38 females, aged 19–52 years, from University of Ilorin. The same group of assessors evaluated all the six batches. Each 10 g sample was served on coded plates, and six samples corresponding to the six treatment combinations were evaluated by each panelist. Before the sensory analysis, assessors were briefed on the sensory attributes and evaluation procedures. Between samples, water and unsalted crackers were provided to cleanse their palates.

### 2.11. Statistical analysis

The experiment followed a 3 (fat type) × 2 (cooking temperature) factorial arrangement in a completely randomized design. Data were analyzed using the MIXED procedure of the Statistical Analysis System (SAS 9.2; SAS Institute Inc., Cary, NC, USA). The experiment was conducted with six replicates. The model included fat type, roasting temperature, and their interaction as fixed effects, while batches were treated as a random effect. For sensory data, assessors were also treated as random effects. A significance level of *P* < 0.05 was used, and least-square means were compared using the PDIFF option. Principal component analysis (PCA) was performed using XLSTAT software (Addinsoft, New York, NY, USA).

## 3. Results and discussion

### 3.1. Fatty acid profile

The FA composition of beef tallow, palm olein, and soybean oil presents distinct variations in SFA, MUFA, and PUFA (Table 2), which are critical factors influencing the nutritional and oxidative stability of the fats used in sausage formulations. BT had the highest SFA content followed by the PO. The predominant SFAs in BT were C16:0 and C18:0. PO had a notably higher palmitic acid content compared to BT and SO. Both BT and PO had higher levels of MUFA mainly C18:1n-9 compared with SO. Soybean oil was the most PUFA-rich fat, predominantly containing C18:2n-6 (48,733 mg/100 g) and C18:3n-3 (6,499 mg/100 g). PO had a moderate PUFA level (12,222 mg/100 g), while beef tallow had the lowest PUFA content (2,813 mg/100 g). Raw beef had lower SFA, MUFA and PUFA compared with the fat sources. The current results are typical of those previously reported for beef tallow [51], soybean oil [52], palm olein [53] and raw beef [54].

**Table 2:** Fatty acid composition of fats and raw beef.

Fatty acid (mg/100 g sample)	Beef tallow	Palm olein	Soybean oil	Raw beef
C12:0	0.00 ± 0.00	485 ± 17.8	0.00 ± 0.00	0.00 ± 0.00
C14:0	3298 ± 140	873 ± 43.21	97.0 ± 6.76	134 ± 8.60
C14:1	679 ± 25.0	0.00 ± 0.00	0.00 ± 0.00	35.0 ± 2.56
C15:0	534 ± 33.4	0.00 ± 0.00	0.00 ± 0.00	0.00 ± 0.00
C16:0	26675 ± 324	35890 ± 330	12125 ± 75.3	1262 ± 20.3
C17:0	1310 ± 89.4	0.00 ± 0.00	194 ± 7.00	0.00 ± 0.00
C18:0	16927 ± 213	4714 ± 149	4074 ± 110	775 ± 10.2
C20:0	97.0 ± 6.90	340 ± 25.6	291 ± 4.20	75.0 ± 5.68
∑SFA	49519 ± 450	42302 ± 390	16781 ± 150	2280 ± 65.2
C16:1	2425 ± 90.2	175 ± 20.2	0.00 ± 0.00	201 ± 9.20
C18:1t11	3686 ± 89.5	0.00 ± 0.00	0.00 ± 0.00	87.0 ± 5.55
C18:1n-7	825 ± 30.3	0.00 ± 0.00	0.00 ± 0.00	0.00 ± 0.00
C18:1n-9	37054 ± 276	42195 ± 330	24444 ± 230	2149 ± 98.6
C21:1	194 ± 5.60	0.00 ± 0.00	0.00 ± 0.00	0.00 ± 0.00
∑MUFA	44184 ± 404	42370 ± 389	24444 ± 200	2437 ± 58.9
C18:2n-6	1067 ± 43.2	11931 ± 120	48733 ± 368	133 ± 11.2
C18:3n-3	776 ± 10.5	291 ± 10.4	6499 ± 120	43.0 ± 5.22
C20:4n-6	970 ± 22.3	0.00 ± 0.00	0.00 ± 0.00	24.0 ± 3.30
C20:5n-3	0.00 ± 0.00	0.00 ± 0.00	0.00 ± 0.00	11.5 ± 1.55
C22:5n-3	0.00 ± 0.00	0.00 ± 0.00	0.00 ± 0.00	23.0 ± 3.18
C22:6n-3	0.00 ± 0.00	0.00 ± 0.00	0.00 ± 0.00	5.50 ± 1.00
∑PUFA	2813 ± 78.2	12222 ± 150	55232 ± 367	240 ± 23.21
∑FA	96516 ± 420	96894 ± 489	96457 ± 454	4957 ± 111
n-3	776 ± 21.4	291 ± 11.2	6499 ± 67.5	83.0 ± 9.89
n-6	2037 ± 88.3	11931 ± 110	48733 ± 202	157 ± 10.2
n-6/n-3	2.63 ± 0.34	41.0 ± 4.56	7.50 ± 0.38	1.89 ± 0.34
PUFA/SFA	0.06 ± 0.01	0.29 ± 0.04	3.29 ± 0.20	0.11 ± 0.03

FA, fatty acid. SFA, saturated fatty acid. MUFA, monounsaturated fatty acid. PUFA, polyunsaturated fatty acid. ∑FA = ∑SFA + ∑MUFA + ∑PUFA

The FA composition of beef sausages containing different fats and roasted at different temperatures is presented in Table 3. The FA profiles of the sausages largely reflected the FA composition of the individual fats used in the formulation. Sausages made with BT and PO had higher levels of SFA and MUFA, while sausages made with SO had significantly higher levels of PUFA, particularly n-6 fatty acids (*P* < 0.05). The SO sausages also had the highest PUFA/SFA and n-6/n-3 ratios, indicating a higher degree of unsaturation, which makes them more prone to oxidation. These findings are consistent with previous studies, which demonstrated that the FA composition of beef sausages [5,6,55,56] lamb sausages [57] and pork sausages [58, 59] mirrored the FA profile of the fats added during processing. Roasting at 240°C generally resulted in higher PUFA and MUFA content, while roasting at 180°C showed higher SFA levels (*P* < 0.05). This suggests that the 240^o^C may reduce thermal degradation of PUFA and MUFA potentially due to the shorter cooking time (15 min) minimizing prolonged exposure to heat. Consistently, mutton grilled at 230 °C for 20 min had higher PUFA and MUFA levels and lower SFA levels than the mutton microwaved at 190 °C for 25 min [60].

**Table 3:** Fatty acid composition of beef sausages containing different fats and roasted at different temperatures.

	Fat type (FT)			Roasting Temperature (RT)			*P* value		
Fatty acid (mg/100 g sausage)	Beef tallow	Palm olein	Soybean oil	180 °C	240 °C	SEM	FT	RT	FT × RT
C12:0	0.00b± 0.00	27.0a ± 2.45	0.00b ± 0.00	6.90 ± 0.34	6.30 ± 2.00	2.01	0.022	0.122	0.211
C14:0	639a ± 23.50	506b ± 30.2	464b ± 28.3	552 ± 30.2	496 ± 30.5	30.8	0.090	0.231	0.110
C14:1	158a ± 10.30	119b ± 9.23	119b ± 5.60	133 ± 7.75	120 ± 6.52	10.1	0.102	0.092	0.234
C15:0	29.0a ± 2.40	0.00b ± 0.00	0.00b ± 0.00	11.5 ± 0.87	8.40 ± 2.30	3.00	0.040	0.205	0.200
C16:0	5794b±104.3	6296a±88.7	5225c±90.4	5134a ± 97	3780b ± 40.2	68.9	0.030	0.020	0.435
C17:0	72.0a ± 5.60	0.00c ± 0.0	11.3b ± 0.78	27.6 ± 3.20	27.3 ± 3.33	5.03	0.033	0.101	0.213
C18:0	4034a ± 94.6	2921c ± 67.5	3110b ± 60.6	4977a ± 67.4	4221b ± 72.3	66.3	0.010	0.032	0.211
C20:0	263 ± 14.5	277 ± 14.00	275 ± 15.9	278 ± 16.2	252 ± 15.3	0.51	0.212	0.109	0.236
∑SFA	10989a ± 209	10145b ±190	8928c ± 140	10488a±150	8982b ± 155	98.6	0.020	0.029	0.540
C16:1	821a ± 67.3	700b ± 43.2	576c ± 56.2	741 ± 45.2	697 ± 40.7	34.5	0.022	0.211	0.367
C18:1t11	500a ± 45.7	299b ± 34.2	299b ± 25.9	350 ± 31.7	357 ± 26.5	20.2	0.012	0.111	0.213
C18:1n-7	45.0a ± 6.44	0.00b ± 0.00	0.00b ± 0.00	13.80 ± 1.20	12.6 ± 0.87	4.11	0.010	0.125	0.126
C18:1n-9	8789b ± 110	9689a ± 87.3	8496c ± 110	8740b ± 121	9303a ± 130	88.0	0.030	0.012	0.102
C21:1	11.25a ± 1.22	0.00b ± 0.00	0.00b ± 0.00	4.60 ± 0.34	4.20 ± 0.55	1.01	0.034	0.342	0.100
∑MUFA	10132b ± 198	10685a ±103	9371c ± 140	9844b ± 120	10370a ± 162	92.0	0.011	0.038	0.281
C18:2n-6	515c ± 20.9	1107b ± 35.7	3337a ± 42.3	1313b ±56.2	1743a ± 48.3	51.7	0.010	0.021	0.155
C18:3n-3	189b ± 10.2	164b ± 9.23	502a ± 18.9	276 ± 17.5	277 ± 10.33	15.8	0.112	0.109	0.314
C20:4n-6	135a ± 9.95	81.0b ± 6.74	81.0b ± 4.56	101 ± 5.67	92.4 ± 5.00	0.22	0.020	0.453	0.322
C20:5n-3	40.5 ± 3.22	40.5 ± 2.55	40.5 ± 3.44	41.4 ± 3.00	37.8 ± 3.10	3.15	0.210	0.333	0.413
C22:5n-3	78.8 ± 4.56	78.8 ± 7.81	78.8 ± 6.20	78.2 ± 4.30	75.6 ± 4.34	4.23	0.230	0.332	0.213
C22:6n-3	20.3 ± 1.44	20.3 ± 1.66	20.3 ± 1.67	20.7 ± 2.00	18.9 ± 1.65	2.44	0.310	0.117	0.413
∑PUFA	977c ± 88.20	1490b ± 80.6	4059a ± 129	1822b ± 90.2	2274a ± 76.2	56.5	0.014	0.010	0.210
∑FA	22098 ± 341	22320 ± 306	22358 ± 350	22154a ± 320	21626b ± 350	187	0.145	0.015	0.213
∑n-3	329b ± 34.0	302b ± 28.9	639a ± 34.2	416 ± 30.3	410 ± 31.4	23.7	0.030	0.320	0.342
∑n-6	650c ± 45.4	1188b ± 50.2	3420a ± 60.2	1497b ± 54.3	1968a ± 54.2	58.3	0.010	0.010	0.232
n-6/n-3	1.98c ± 0.09	3.94b ± 0.22	5.35a ± 0.34	3.59b ± 0.78	4.80a ± 0.44	0.81	0.020	0.021	0.452
PUFA/SFA	0.09c ± 0.01	0.15b ± 0.05	0.45a ± 0.04	0.17b ± 0.04	0.26a ± 0.04	0.05	0.010	0.021	0.221

a, b, c, means with different superscripts in a row are significantly different (*P* < 0.05). SEM, standard error of mean. FA, fatty acid. SFA, saturated fatty acid. MUFA, monounsaturated fatty acid. PUFA, polyunsaturated fatty acid. ∑FA = ∑SFA + ∑MUFA + ∑PUFA

### 3.2. Cholesterol and Cholesterol oxides

The cholesterol content and cholesterol oxides in beef sausages made with different fats and roasted at various temperatures are shown in Table 4. The BT sausages had higher (*P* < 0.05) cholesterol content than the SO and PO sausages. Beef tallow naturally contains high levels of cholesterol because it is an animal-based fat source. The addition of SO and PO effectively dilutes the cholesterol content in the sausages because they contribute fats without adding cholesterol. Likewise, the partial or total replacement of beef tallow with safflower oil reduced cholesterol content in wieners [10]. In addition, replacing lard with vegetable oils lowered cholesterol content in emulsion-type pork sausages [58]. Beef sausages roasted at 180^o^C had lower cholesterol content than those roasted at 240^o^C (*P* < 0.05). High-temperature cooking causes significant water evaporation, concentrating the remaining nutrients, including cholesterol, in the final product. This aligns with the lower water content in the 240 °C sausages compared with those roasted at 180^o^C. Moreover, roasting at 180^o^C was performed over a longer duration (30 min), which might enhance cholesterol migration and loss. Also, it is possible that part of the cholesterol in the 180^o^C sausages have been converted into oxidized derivatives. Similarly, higher internal-end temperature increased cholesterol content in beef patties [61] and turkey [62].

**Table 4:** Cholesterol and Cholesterol oxides in beef sausages containing different fats and roasted at different temperature.

	Fat type (FT)			Roasting temperature (RT)		SEM	P value		
Item	Beef tallow	Palm olein	Soybean oil	180^o^C	240^o^C		FT	RT	FT x RT
Cholesterol (mg/100 g sausage)	46.23a ± 2.23	32.98b ± 2.50	32.85b ± 2.06	36.23b ± 3.05	38.38a ± 3.00		<.001	0.012	0.921
Cholesterol oxidation products (µg/100 g sausage)									
7-ketocholesterol	44.24 ± 2.00	62.75 ± 3.21	101.9 ± 4.56	120.09±5.20	19.14 ± 1.10	1.47	<.001	<.001	<.001
7β –hydroxycholesterol	9.79 ± 0.78	10.84 ± 0.76	26.86 ± 2.56	4.74 ± 0.17	26.92 ± 2.17	1.18	<.001	<.001	<.001
7α–hydroxycholesterol	9.17 ± 0.67	13.51 ± 1.21	3.07 ± 0.44	4.00 ± 0.24	13.17 ± 1.20	0.58	<.001	<.001	<.001
7-hydroperoxycholesterol	24.14 ± 1.32	1.70 ± 0.03	23.02 ± 1.36	1.53 ± 0.13	31.04 ± 2.22	1.43	<.001	<.001	<.001
25-hydroxycholesterol	11.65 ± 1.00	5.69 ± 0.64	11.07 ± 0.66	15.46 ± 0.78	3.49 ± 0.32	0.50	0.001	<.001	<.001
Cholesta-3,5 dien-7-one	1.08 ± 0.02	3.21 ± 0.22	16.82 ± 1.76	12.99 ± 1.20	1.08 ± 0.11	0.73	<.001	<.001	<.001
5,6β-epoxycholesterol	2.25 ± 0.02	17.3 ± 1.20	73.56 ± 3.46	59.57 ± 3.15	2.50 ± 0.32	1.62	<.001	<.001	<.001
6β-epoxide	1.45 ± 0.04	3.45 ± 0.54	3.75 ± 0.30	4.27 ± 0.15	1.50 ± 0.12	0.34	<.001	<.001	<.001
19–hydroxycholesterol	1.50 ± 0.02	73.34±4.23	1.50 ± 0.12	1.50 ± 0.10	49.4 ± 3.44	0.47	0.015	<.001	<.001
22R -hydroxycholesterol	104.14± 3.25	19.89 ±1.09	1.25 ± 0.10	78.78 ± 3.45	0.53 ± 0.05	1.14	<.001	<.001	<.001
20α-hydroxycholesterol	0.50 ± 0.01	3.95 ± 0.36	0.55 ± 0.06	0.53 ± 0.03	2.80 ± 0.03	0.12	<.001	<.001	<.001
Total COP	164.96± 4.23	148.47±4.56	263.38±6.78	244.45±5.40	140.09±4.21	10.23	<.001	<.001	<.001

a,b,c means with different superscripts along a row for each factor are significantly different (*P* < 0.05). SEM, standard error of mean.

The interaction between fat type and roasting temperature on COPs was significant, with the specific details provided in Table 5. The SO-180 sausages had higher levels of 7-ketocholesterol compared to the other treatments, while in SO-240 sausages, the levels significantly decreased (*P* < 0.05). The BT and PO sausages exhibited moderate levels of 7-ketocholesterol, which also decreased at 240°C (*P* < 0.05). The SO-240 sausages had higher levels of 7β-hydroxycholesterol compared to the other treatments (*P* < 0.05), and the PO-240 sausages showed higher 7β-hydroxycholesterol than the PO-180 sausages (*P* < 0.05). There was no difference in 7β-hydroxycholesterol levels between the BT-180 and BT-240 sausages (*P* > 0.05). The PO-240 sausages had significantly higher 7α-hydroxycholesterol compared to the other treatments (*P* < 0.05), while both PO and BT sausages showed lower values at their respective temperatures. The levels of 7α-hydroxycholesterol in PO-180 and PO-240 sausages were similar. The BT-240 and SO-240 sausages had higher concentrations of 7-hydroperoxycholesterol, whereas the PO sausages showed relatively stable and lower levels across temperatures (*P* < 0.05). The SO-180 and BF-180 sausages had higher levels of 25-hydroxycholesterol, while the PO-180 sausages produced significantly lower amounts (*P* < 0.05). The SO-180 sausages had the highest levels of cholesta-3,5-dien-7-one and 5,6β-epoxy cholesterol (*P* < 0.05), while BT and PO had lower and consistent levels across temperatures. For 6β-epoxide and 19-hydroxycholesterol, the PO-240 sausages had higher levels, while the BT and SO sausages remained lower and stable across temperatures. The BT-180 sausages showed higher concentrations of 22R-hydroxycholesterol, whereas the other fats and temperatures resulted in much lower amounts (*P* < 0.05). The SO-180 sausages had the highest total COP, while the BT-180 and PO-240 sausages exhibited relatively lower amounts.

**Table 5:** Details of interaction between fat type and roasting temperature on the cholesterol oxidation products of beef sausages.

Cholesterol oxides (µg/100 g sausage)	Beef tallow		Palm olein		Soybean oil	
	180^O^C	240^O^C	180^O^C	240^O^C	180^O^C	240^O^C
7-ketocholesterol	83.23b	5.24d	82.26b	41.25c	190.80a	10.95d
7β –hydroxycholesterol	14.21bc	5.36 cd	2.00d	21.68b	2.00d	53.72a
7α–hydroxycholesterol	11.06b	7.28bc	0.50d	26.53a	0.45d	5.69c
7-hydroperoxycholesterol	1.45b	46.83a	1.65b	1.75b	1.49b	44.54a
25-hydroxycholesterol	19.00a	4.31b	5.83b	5.56b	21.55a	0.60c
Cholesta-3,5 dien-7-one	1.15b	1.00b	5.32b	1.10b	32.49a	1.15b
5,6β-epoxycholesterol	2.50c	2.50c	32.10b	2.50c	144.62a	2.50c
6β-epoxide	1.50b	1.50b	5.41a	1.50b	6.00a	1.50b
19–hydroxycholesterol	1.50b	1.50b	1.50b	145.19a	1.50b	1.50b
22R -hydroxycholesterol	201.05a	7.22c	33.78b	6.00c	1.50c	1.00c
20α-hydroxycholesterol	0.50b	0.50b	0.55b	7.35a	0.55b	0.55b
Total	251.92b	78.00e	79.27e	217.66c	402.15a	124.6d

a,b,c means with different superscripts along a row are significantly different (*P* < 0.05).

The lowest total COPs were observed at 240°C across all fat types. The BT sausages, being rich in SFA, resulted in lower COP across all temperatures. However, specific cholesterol oxides, such as 22R-hydroxycholesterol, were elevated at 180°C. The PO sausages showed intermediate oxidation levels, with significant increase at 240°C for some cholesterol oxides, reflecting its balance of saturated and unsaturated FA. From this, it could be inferred that the SO sausages produced the highest levels of most cholesterol oxides, particularly at 180°C, due to their high PUFA content and the longer cooking duration (30 min), which made them more susceptible to oxidation. Previous studies on horse meat [39] and eggs [63] posited that a higher degree of lipid unsaturation promotes cholesterol oxidation by creating a pro-oxidant environment with the presence of free radicals and hydroperoxides [64]. Furthermore, higher PUFA levels led to an increase in cholesterol oxides in sardines [65], while greater unsaturation in a model system was shown to enhance the formation of cholesterol oxides [66].

Roasting at 180^o^C for 30 min led to higher cholesterol oxides, especially in the SO sausages. it seems the extended time allowed for more gradual oxidation, particularly affecting PUFA in SO. Roasting at 240°C for 15 min reduced total cholesterol oxides for all BT and SO sausages. This suggests that although the temperature was higher, the shorter cooking time limited the extent of oxidation or thermal degradation of cholesterol. This finding aligns with Alina et al. [67], who reported higher levels of cholesterol oxides in mutton microwaved at 190°C for 25 min compared to those grilled at 230°C for 20 min. In addition, prolonged heating at high temperature enhances cholesterol oxides in a model system [66,68].

### 3.3. Lipid and protein oxidation

Sausages containing SO had significantly higher TBARS values (P < 0.05) compared to the BT and PO sausages (). This can be attributed to the higher PUFA content in the SO sausages, as PUFAs are more susceptible to lipid oxidation due to the presence of multiple double bonds. A similar trend was observed in previous studies, where the partial or total replacement of beef tallow with safflower oil led to an increase in TBARS values in wieners [10]. Likewise, replacing back fat with fish and olive oils enhanced TBARS in frankfurters [11], further confirming the role of PUFAs in accelerating lipid oxidation. Although not statistically significant (P = 0.063), there was a noticeable trend indicating that sausages cooked at 180°C had slightly higher TBARS values than those roasted at 240°C. This suggests that higher roasting temperatures may mitigate lipid oxidation, potentially due to shorter cooking times minimizing prolonged exposure to heat. However, rapid cooking at higher temperatures could limit oxidative degradation by reducing overall exposure time, thereby preserving unsaturated fatty acids. In addition, it is possible that the higher temperature induces the formation of Maillard reaction products, some of which possess antioxidant properties that may help slow lipid oxidation. Similarly, beef patties roasted at 150°C and 190°C for 10 min had higher TBARS values than those roasted at 230°C, 270°C, and 310°C for the same duration [69]. In addition, pork grilled at 190°C for 2 min had higher TBARS levels than pork roasted at 150°C for 20 min [70].

Despite significant changes in lipid oxidation, protein oxidation remained unaffected by fat type and roasting temperature. This could be due to the protective effect of certain antioxidants in the beef sausage, such as the garlic and ginger powder, which help prevent oxidative damage to proteins. Moreover, while lipid oxidation products such as aldehydes and ketones can promote protein oxidation, the levels of MDA observed in this study may not have been high enough to cause a measurable impact. Contrarily, protein carbonyls in beef patties increased as roasting temperature increased from 150–310 °C [68].

### 3.4. Chemical composition

The type of fat did not significantly influence the chemical composition of beef sausages (Table 7). The effect of fat type on the chemical composition of sausages varies across the literature. For instance, replacing beef tallow with palm kernel oil did not alter the chemical composition of fermented beef sausage [55]. The partial or total replacement of beef tallow with safflower oil did not affect the chemical composition of wieners [11]. Similarly, substituting lard with olive oil did not affect the moisture, fat, and protein content of lamb sausage [57]. However, replacing lard with linseed and chia seed oils reduced the moisture, ash, protein, and fat content of lamb sausage [57]. These discrepancies in outcomes may be attributed to differences in fat types and levels, as well as variations in meat types and sausage formulations.

Beef sausages roasted at 180°C had higher water and crude fat levels than those roasted at 240°C (*P* < 0.05). It seems the lower temperature causes a slower cooking process, allowing the sausages to retain more water and fat. The slower heat transfer results in less rapid evaporation of water and prevents the excessive melting and dripping of fat. Despite the longer cooking time, the moderate temperature preserves the structural integrity of the sausage, minimizing water and fat loss. Conversely, at 240°C, the higher temperature causes rapid water evaporation and greater fat loss due to faster melting and dripping of fat from the sausage. Also, the higher temperature may increase protein shrinkage, which further expels water and fat from the sausages. This results in lower water and fat content in sausages roasted at 240°C. Consistently, increased grilling temperature resulted into higher water loss in lamb patties [26].

### 3.5. Physicochemical properties

The color coordinates, cook loss, and pH of beef sausages containing different fats and cooked at varying temperatures are outlined in Table 7. Neither fat type nor temperature affected the pH of the sausages (*P* > 0.05). This suggests that the acid-base balance of the sausage matrix remains stable regardless of these variables. This observation partly aligns with that of Xia et al. [67], who reported that beef patties roasted at 190–310°C had similar pH values, which were higher than those of beef patties roasted at 150°C.

**Table 7:** Chemical composition and physicochemical properties of beef sausages containing different fats and roasted at different temperatures.

	Fat types (FT)							Roasting temperature (RT)			SEM			*P* value				
Chemical composition (%)	Beef tallow		Palm olein		Soybean oil			180^o^C		240^o^C				FT		RT		FT x RT
Ether extract	22.42±0.50		22.72±0.67		22.81±0.44			23.03a ± 0.64		22.34b ±0.36	0.28			0.537		0.037		0.789
Ash	2.80±0.23		2.71±0.22		2.70±0.15			2.80±0.34		2.68±0.55	0.23			0.908		0.550		0.468
Physicochemical traits																		
pH	5.86 ± 0.34		5.85 ± 0.24		5.88 ± 0.34			5.86 ± 0.53		5.85 ±0.34	0.12			0.124		0.345		0.200
L*	33.52 ±3.92		34.32±2.16		35.83±1.91			30.40±2.34		38.61±3.22	0.62			0.074		<0.001		<0.001
a*	7.00 ± 1.01		10.21 ± 1.10		11.50 ± 1.10			7.63 ± 1.00		11.51±1.21	0.24			<0.001		<0.001		<0.001
b*	18.20 ± 2.13		14.90 ± 2.16		15.61 ± 1.59			18.02 ± 2.34		14.52 ±1.56	0.76			0.023		0.001		0.001
Cook loss (%)	8.75±0.95		6.75±0.85		6.50 ±0.56			8.51 ± 0.89		6.17±0.77	0.52			0.017		0.001		0.001

a,b, means with different superscripts along a row for each factor are significantly different (*P* < 0.05). SEM, standard error of mean.

The interaction between fat type and cooking temperature was significant for both color and cook loss, as detailed in Table 8. Lightness increased significantly in the BT sausages when roasted at 240°C compared to 180°C. No significant changes in lightness were observed in sausages with PO or SO between 240°C and 180°C. The increase in the lightness of the BT-240 sausages may be due to the higher melting point of tallow, which solidifies and reflects more light after roasting at higher temperatures. In contrast, PO and SO, with lower melting points, do not undergo the same structural changes, resulting in consistent lightness across temperatures.

**Table 8:** Details of interaction between fat type and roasting temperature on color and cook loss in beef sausages.

Item	Beef tallow		Palm olein		Soybean oil	
	180^o^C	240^o^C	180^o^C	240^o^C	180^o^C	240^o^C
L*	24.1d	42.8a	33.3c	35.3bc	33.7bc	37.8b
a*	2.9c	11.0ab	9.5b	10.9b	10.5b	12.6a
b*	22.6a	13.7b	15.2b	14.7b	16.2b	15.1b
Cook loss (%)	11.50a	6.00b	8.00b	5.50b	6.00b	7.00b

a,b,c,d means with different superscripts along a row are significantly different (*P* < 0.05).

Redness increased significantly in BT and SO sausages when roasted at 240°C compared to 180°C, while the redness of PO sausages did not differ between the two temperatures. The significant increase in the redness of the BT-240 and SO-240 sausages is likely caused by more intense Maillard reactions and pigment concentration as water evaporates at higher temperatures. Palm olein sausages do not show this change, possibly due to a different FA composition that influences color stability under heat.

Yellowness and cook loss decreased significantly in BT sausages when roasted at 240°C compared to 180°C, but both parameters remained similar in PO and SO sausages regardless of roasting temperature. This may be due to the higher fat retention and reduced water evaporation of beef tallow compared to the other oils. The stability in yellowness and cook loss in PO and SO sausages suggests that these fats have a lower impact on structural changes during cooking, maintaining consistent properties across temperatures.

### 3.6. Textural profile

The textural properties of beef sausages made with different fat types and cooked at two different temperatures are presented in Table 9. The BT sausages had the highest hardness, followed by the PO and SO sausages. These results appear to follow the trend of SFA content in the sausages. Beef tallow has a higher SFA content, which tends to solidify more at cooking temperatures, contributing to a firmer texture. In contrast, palm olein and soybean oil, which have higher levels of unsaturated FAs, resulted in softer sausages. A similar trend was observed in the springiness, gumminess and chewiness of beef sausages. Saturated fats like beef tallow may retain elasticity better during cooking, providing a more resilient texture. Gumminess is related to hardness and cohesiveness, so the higher gumminess of the BT sausages may be linked to their firmer, more cohesive structure due to the higher saturation of the fat. Chewiness is related to the combination of hardness, cohesiveness, and springiness. As with gumminess, the higher chewiness in beef tallow sausages likely results from its higher SFA content, which contributes to a firmer, more resilient texture. Similar to our findings, replacing beef tallow with palm kernel oil lowered the hardness, chewiness, and gumminess of fermented sausages [57]. Further, replacing lard with emulsified vegetable oils reduced the hardness, chewiness, and cohesiveness of deer sausages [8].

**Table 9:** Textural profile of beef sausage containing different fats and roasted at different temperature.

	Fat types (FT)			Roasting temperature (RT)		SEM	*P* value		
Item	Beef tallow	Palm olein	Soybean oil	180^o^C	240^o^C		FT	RT	FT x RT
Hardness (N)	34.3a±2.45	27.0b ± 2.11	23.8c ±2.00	28.8 ±2.22	28.2 ± 1.98	0.55	<0.001	0.206	0.421
Cohesiveness	0.81 ± 0.15	0.80 ± 0.12	0.80 ± 0.11	0.81 ± 0.12	0.80 ± 0.14	0.01	0.691	0.190	0.448
Springiness	0.81a ±0.10	0.76ab ± 0.21	0.74b ± 0.17	0.78 ± 0.12	0.76 ± 0.12	0.02	0.021	0.425	0.536
Gumminess	28.0a ± 2.17	21.7b ± 2.44	19.0c ± 2.11	22.9 ± 2.78	22.8 ± 2.80	0.52	<0.001	0.731	0.835
Chewiness (N)	22.7a ± 3.21	16.3b± 1.77	14.2c ±2.33	17.9 ± 2.16	17.5 ±2.16	0.48	<0.001	0.343	0.735

a,b,c means with different superscripts along a row for each factor are significantly different (*P* < 0.05). SEM, standard error of mean.

### 3.7. Sensory attributes

The sensory attributes of beef sausages prepared with different fat types and cooked at different temperatures were evaluated (). The PO sausages had better taste score than the BT sausages (*P* < 0.05), but similar to the SO sausages. All fat types had similar aroma, tenderness and juiciness scores (*P* > 0.05). The SO sausages had higher appearance score than the BT sausages (*P* < 0.05). The appearance score in the PO sausages did not differ from that of BT and SO sausages. The PO and SO sausages had greater overall acceptance scores than the BT sausages (*P* < 0.05). Likewise, the partial or total replacement of lard with pumpkin and melon oils did not impair the sensory quality of deer sausages [8] while the partial or total replacement of beef tallow with safflower oil did not affect the sensory attributes of wieners [10].

Roasting temperature did not significantly affect the aroma, taste, tenderness and overall acceptance scores in beef sausages (*P* > 0.05). Beef sausages roasted at 180^o^C for 30 min had greater appearance and juiciness scores than the sausages cooked at 240^o^C for 15 min (*P* < 0.05). The longer cooking time at a lower temperature retained more water and fat as evident in the chemical composition data.

### 3.8. Principal component analysis

**Fig 1:**
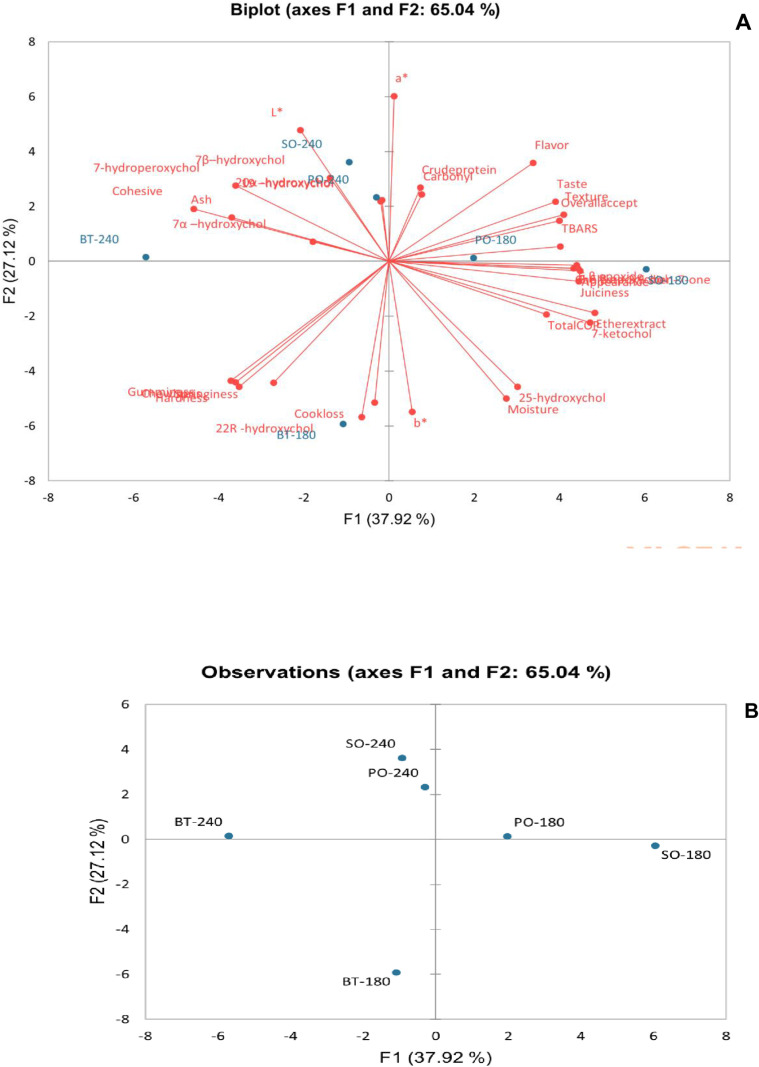
Principal component analysis (PCA) of the characteristics of beef sausages containing different fat types and cooked at different temperatures. (A) PCA biplot showing the relationship between variables and observations. **(B)** PCA score plot of observations along principal components. BF-180, Beef sausages containing 20% beef tallow and roasted at 180^o^C for 30 min. BF-240, Beef sausages containing 20% beef tallow and roasted at 240^o^C for 15 min. PO-180, Beef sausages containing 20% palm olein and roasted at 180^o^C for 30 min. PO-240, Beef sausages containing 20% palm olein and roasted at 240^o^C for 15 min. SO-180, Beef sausages containing 20% soybean oil and roasted at 180^o^C for 30 min. SO-240, Beef sausages containing 20% soybean oil and roasted at 240^o^C for 15 min.

PCA was performed to visualize how fat type and roasting temperature influence sausage quality characteristics by reducing the dimensionality of the dataset while retaining the most important variance-driving factors. The first two principal components (PC1 and PC2) explain 65.04% of the total variance, meaning they capture the most important differences between treatments. PC1 (37.92%) is largely driven by variations in cholesterol oxides, fat content, and sensory attributes (Fig 1A), while PC2 (27.12%) is associated with oxidation markers, water, textural properties (hardness, chewiness, springiness), and yellowness (Fig 1A).

Sausages cooked at 180 °C, particularly BT-180 and SO-180, are positioned far from the others (Fig 1B), indicating that they are distinct in terms of higher cholesterol oxides, fat content, and sensory attributes like juiciness and appearance. This is because beef sausages roasted at 180°C for 30 min retained more fat and water, leading to higher juiciness, flavor intensity, and a softer texture. The extended cooking time promoted gradual Maillard reactions and lipid oxidation, enhancing the development of desirable flavors while also increasing COPs. The slower cooking process resulted in a darker appearance due to continuous browning reactions, reducing lightness. Conversely, sausages cooked at 240 °C cluster together (Fig 1B), and are characterized by increased lightness, and hardness and reduced TBARS and selected COPs. Sausages roasted at 240°C for 15 min experienced more rapid fat loss, reducing overall juiciness and fat content. The high temperature accelerated water evaporation, making the sausages firmer and drier. Excessive heat may lead to some charring, which may have reduced flavor complexity. However, the shorter cooking time limited the extent of oxidation reactions, resulting in lower COPs and TBARS compared to the longer, lower-temperature cooking method. Furthermore, the PCA highlights key contrasts between groups of sausages. Sausages that score higher in flavor, taste, texture, overall acceptance, and redness tend to differ from those with higher cook loss, hardness, springiness, and gumminess, emphasizing that roasting and fat type influence sensory characteristics. In addition, different cholesterol oxidation products associate with different treatments, suggesting that roasting temperature plays a role in cholesterol oxidation pathways. For instance, sausages exhibiting higher lightness, 7β–hydroxycholesterol, 7α–hydroxycholesterol, 7-hydroperoxycholesterol, and 20α-hydroxycholesterol have attributes that contrast those showing higher water, fat content, total cholesterol oxides, 7-ketocholesterol, 25-hydroxycholesterol, 6β epoxide and juiciness. Overall, these findings illustrate how the interplay between fat type and roasting conditions impacts the physicochemical and sensory properties of beef sausages, providing insights into optimizing processing conditions to balance oxidative stability and sensory acceptability.

## Conclusion

This study demonstrated that fat type and roasting temperature significantly affected the FA composition, cholesterol oxides, physicochemical properties and sensory properties of beef sausages. Roasting at 240°C increased total cholesterol, PUFA and MUFA levels, and reduced SFA levels. Roasting at 240^o^C increases lightness and redness but decreases yellowness and cook loss, particularly in sausages with beef tallow. The SO and PO sausages had less pronounced changes in color, and cook loss compared to the BT sausages. Sausages made with soybean oil were rich in PUFA, but had higher oxysterols and TBARS values, especially at 180°C. Beef tallow sausages exhibited higher total cholesterol, lower oxysterol and greater stability, but scored lower in sensory attributes like taste and overall acceptance. Palm olein sausages provided a balance, with moderate cholesterol oxidation levels, favorable sensory scores, and good overall acceptance. Roasting at 240^o^C can mitigate oxysterol levels in PUFA-rich sausages while altering sensory and physicochemical properties, offering practical recommendations for improving product safety and quality. Future research on the effects of varying cooking methods and temperatures on both oxidative stability and sensory attributes would provide valuable insights for optimizing sausage production.

## Consent to participate

Informed written consent was obtained from all individuals that participated in the sensory analyses.

**Table 6:** Lipid oxidation and protein oxidation in beef sausage containing different fats and roasted at different temperatures.

	Fat types (FT)			Roasting temperature (RT)		SEM	*P* value		
Item	Beef tallow	Palm olein	Soybean oil	180^o^C	240^o^C		FT	RT	FT x RT
Malondialdehyde (mg/kg sausage)	0.38b ± 0.02	0.47b ± 0.04	0.67a ± 0.05	0.53 ± 0.04	0.46 ± 0.06	0.11	0.048	0.063	0.877
Carbonyl (mmol/mg protein in sausage)	0.22 ± 0.03	0.26 ± 0.03	0.28 ± 0.05	0.24 ± 0.03	0.26 ± 0.04	0.06	0.210	0.144	0.073

a,b, means with different superscripts along a row for each factor are significantly different (*P* < 0.05). SEM, standard error of mean.

**Table 10:** Sensory attributes of beef sausages containing different fats and cooked at different temperatures.

	Fat types (FT)			Roasting temperature (RT)		SEM	*P* value		
Item	Beef tallow	Palm olein	Soybean oil	180^o^C	240^o^C		FT	RT	FT x RT
Taste	6.1b ± 0.3	6.8a ± 0.4	6.7ab ± 0.3	6.6 ± 0.3	6.4 ± 0.4	0.21	0.034	0.405	0.716
Flavor	6.3 ± 0.4	6.6 ± 0.4	6.6 ± 0.3	6.5 ± 0.4	6.4 ± 0.3	0.19	0.344	0.799	0.821
Texture	6.4 ±0.3	6.9 ± 0.5	6.7 ± 0.4	6.8 ±0.5	6.6 ± 0.4	0.19	0.116	0.347	0.400
Appearance	6.4b ± 0.3	6.9ab ± 0.5	7.0a ± 0.3	7.0^a^ ±0.4	6.5^b^ ± 0.3	0.18	0.037	0.016	0.265
Juiciness	6.3 ± 0.5	6.7 ± 0.4	6.8 ± 0.4	6.8^a^ ± 0.4	6.3^b^ ± 0.4	0.20	0.142	0.014	0.584
Overall acceptance	6.4b ± 0.2	7.3a ± 0.3	7.1a ± 0.5	7.1 ± 0.4	6.7 ± 0.3	0.19	0.003	0.114	0.114

a,b, means with different superscripts along a row for each factor are significantly different (*P* < 0.05). SEM, standard error of mean. 9 = like extremely, 8 = like very much, 7 = like moderately, 6 = like slightly, 5 = neither like nor dislike, 4 = dislike slightly, 3 = dislike moderately, 2 = dislike very much, and 1 = dislike extremely
